# Fokker–Planck dynamics of the El Niño-Southern Oscillation

**DOI:** 10.1038/s41598-020-73449-7

**Published:** 2020-10-01

**Authors:** Soon-Il An, Soong-Ki Kim, Axel Timmermann

**Affiliations:** 1 Yonsei University, 50 Yonsei-ro, Seodaemun-gu, Seoul, 03722 South Korea; 2 Center for Climate Physics, Institute for Basic Science (IBS), Busan, 46241 South Korea; 3 Pusan National University, Busan, 46241 South Korea

**Keywords:** Climate change, Physical oceanography, Applied mathematics

## Abstract

The asymmetric nature of the El Niño-Southern Oscillation (ENSO) is explored by using a probabilistic model (PROM) for ENSO. Based on a Fokker–Planck Equation (FPE), PROM describes the dynamics of a nonlinear stochastic ENSO recharge oscillator model for eastern equatorial Pacific temperature anomalies and equatorial Pacific basin-averaged thermocline depth changes. Eigen analyses of PROM provide new insights into the stationary and oscillatory solutions of the stochastic dynamical system. The first probabilistic eigenmode represents a stationary mode, which exhibits the asymmetric features of ENSO, in case deterministic nonlinearities or multiplicative noises are included. The second mode is linked to the oscillatory nature of ENSO and represents a cyclic asymmetric probability distribution, which emerges from the key dynamical processes. Other eigenmodes are associated with the temporal evolution of higher order statistical moments of the ENSO system. The model solutions demonstrate that the deterministic nonlinearity plays a stronger role in establishing the observed asymmetry of ENSO as compared to the multiplicative stochastic part.

## Introduction

Tropical Pacific sea surface temperature anomalies (SSTA) on interannual timescales are mainly controlled by the El Niño-Southern Oscillation (ENSO) phenomenon. They can trigger changes in the large-scale atmospheric circulations, which impact weather, ecosystems and societies. The typical evolution of ENSO involves an early spring initiation, a peak boreal winter warming and a phase transition to La Niña conditions during the following summer^1^. A positive feedback process between equatorial trade winds and the zonal equatorial SST gradient [referred to as “*Bjerknes feedback*” (BJ)^2^] leads to growth of ENSO. The alternation between El Niño and La Niña has been explained by the delayed negative feedback through slow oceanic discharge and the Rossby wave adjustment processes. Furthermore, the anomalous accumulation of warm water in the tropical western Pacific is an important pre-cursor of El Niño events^3,4^.

The aforementioned two essential processes can be integrated into the recharge oscillator model (ROM), described by the following equations^4,5^:

1a $${\raise0.7ex\hbox{${d T_{E} }$} \!\mathord{\left/ {\vphantom {{d T_{E} } {dt}}}\right.\kern-\nulldelimiterspace} \!\lower0.7ex\hbox{${dt}$}} = I_{BJ} T_{E} + F\left[ h \right],$$

1b $${\raise0.7ex\hbox{${d\left[ h \right]}$} \!\mathord{\left/ {\vphantom {{d\left[ h \right]} {dt}}}\right.\kern-\nulldelimiterspace} \!\lower0.7ex\hbox{${dt}$}} = - \varepsilon \left[ h \right] - \alpha T_{E} ,$$

where $T_{E}$ and $\left[ h \right]$ represent the equatorial eastern Pacific SST anomaly and the zonal mean equatorial Pacific thermocline depth anomaly over the equatorial Pacific, respectively. $I_{BJ}$ and $\varepsilon$ together determine the collective growth/damping rate ($(I_{BJ} - \varepsilon )/2)$, which is referred to as the *Bjerknes stability index*^6^; the frequency is determined by $\sqrt {\alpha F - \frac{{\left( {I_{BJ} + \varepsilon } \right)^{2} }}{4}}$, which is called the *Wyrtki index*^7^. The cyclic features of ENSO simulated by this linear version of the ROM were limited to be regular and symmetric^5^.

**Figure 1:**
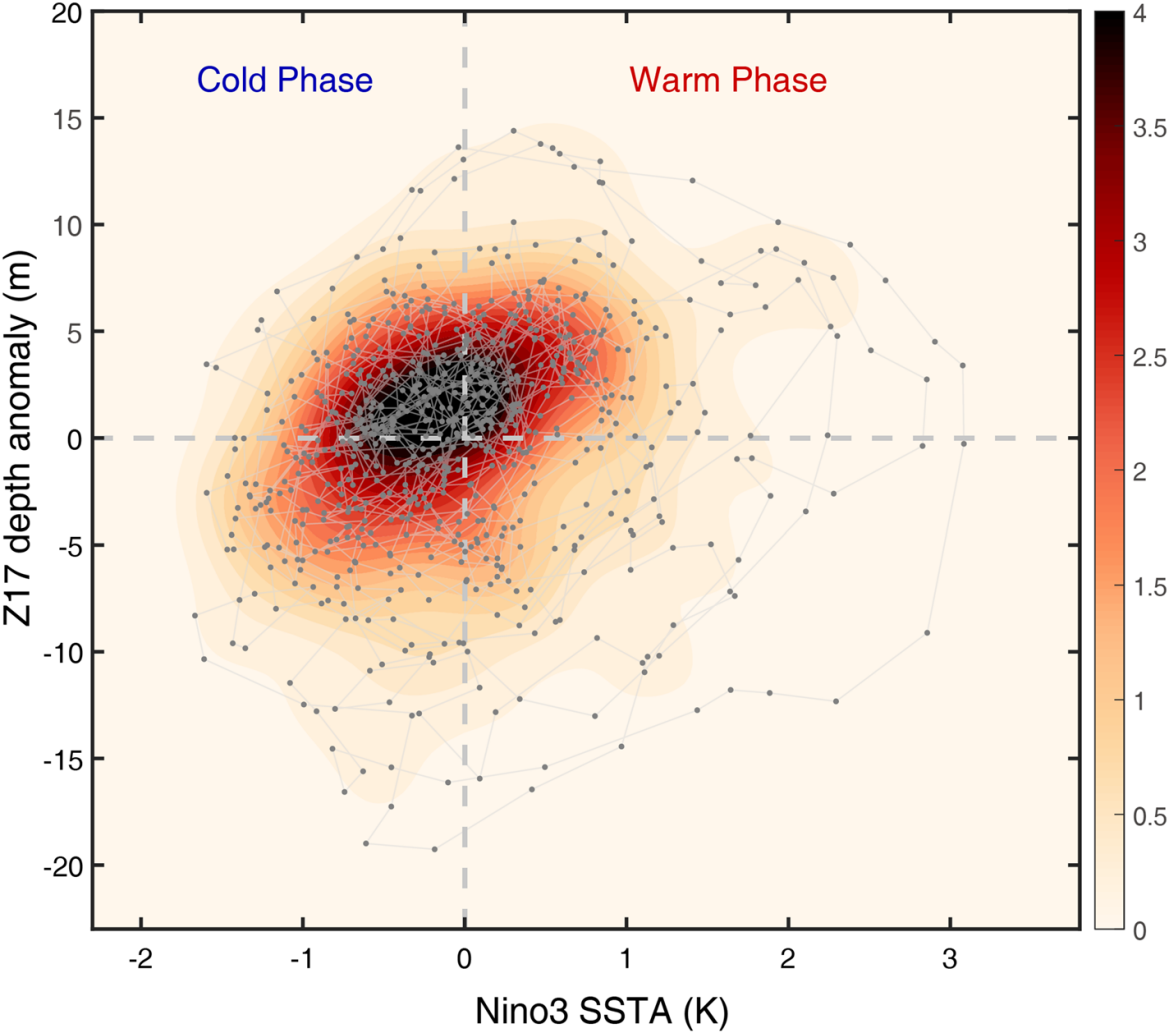
Observed SST-thermocline depth phase diagram for equatorial Pacific. Joint probability density distributions (shading) of monthly-mean Niño-3 SST anomalies (5° S–5° N, 150°–90° W) and equatorial Pacific thermocline depth anomalies, and phase diagram (gray line) depicted by time sequential tracks of these two variables obtained for the period 1958–2016. Thermocline depth anomalies indicated the 17 °C isotherm depth anomalies averaged over the equatorial band between 5° S–5° N, 120° E–80° W. Both variables are smoothed using a 1-2-1 filter. Contour units for probability density are in %.

However, observations show a more complex dynamical evolution of equatorial eastern Pacific SSTA, which is characterized by a positively skewed probability distribution^8,9^ and an asymmetric evolution of the ENSO cycle^10^ (see Fig. 1). Whereas El Niño is usually followed by La Niña, La Niña events are not always followed immediately by El Niño. The termination of El Niño is considerably faster and more deterministic than that of La Niña^11,12^. Historically, different theories have been proposed to mathematically describe the irregularity of ENSO: one considers nonlinear dynamical systems’ theory^13–15^ and the other is based on the notion that ENSO is a essentially a stochastically forced linear system^16–18^. Neither of these approaches explains the nature of the asymmetric stationary probability distribution and other nonlinear oscillatory features (Fig. 1 and also see Fig. 2 of Timmermann et al.^5^).

Recent studies^9–21^ have proposed to extend the linear ROM by including deterministic nonlinearities and additive or multiplicative stochastic forcing. For instance, the linkage between subsurface temperature and thermocline depth anomalies was expressed in terms of a cubic relationship^22^. However, a deterministic system based on the cubic nonlinearities does not produce the observed non-Gaussian behavior seen in the observations^20^. Multiplicative noise, on the other hand, can become a source for non-Gaussian dynamics and the emergence of positively skewed stationary probability density, even in the absence of deterministic nonlinearities. Rather than employing stochastic noise, a constant term can be added into ROM (Eq. 1a), to represent the effect of the Madden–Julian Oscillation. Such simple modifications break the symmetry of a system and can produce reasonable skewness^21^. Furthermore, other studies^23,24^ converted the ROM by adding multiplicative noise to a Fokker–Planck equation (FPE) and then solved it analytically by adopting a higher-order closure. Their analytical solution for the equilibrium probability density function demonstrated that multiplicative noise can break the symmetry of the probability distribution. Another study^25^, explored the bimodality of ENSO and the possibility to generate strong and moderate El Niño events in ROM through nonlinear feedback processes that capture the effect of the SST dependence of convective instability. In their study, strong El Niño events are stochastically triggered, whereas strong La Niña events are mainly driven by strong deterministic discharge following a strong El Niño. This ROM version generated weak SSTA skewness. These theoretical studies showed that the asymmetric stationary probability density distribution of the ENSO system may be explained either by state-dependent noise or by the representation of nonlinear processes. However, these simplified modeling studies did not consider the asymmetric temporal evolution of the probability density.

Here, we transform a nonlinear ROM with both deterministic and stochastic nonlinearities into FPE and study the eigenmode dynamics of the FPE, which provides insights into the temporal evolution of the different moments of the probability distribution. First, nonlinear dynamical heating (NDH) was incorporated. NDH plays a key role in the amplitude asymmetry of ENSO, as it produces a positive SSTA tendency regardless of the ENSO phase^15^. Second, multiplicative SSTA-modulated noise is added. Finally, the nonlinear ROM is then capable of capturing the nonlinear nature of ENSO visualized as both the asymmetric stationary and cyclic probability density distributions. By conducting an eigen analysis of the FPE^26^, as the leading modes, the stationary and cyclic probability density modes are separated. Finally, the roles of the higher modes are also discussed.

## Results

### Observed probability density distribution of ENSO

We estimate the probability density distribution of the observed ENSO system by calculating the 2-dimensional normalized histogram of the Niño-3 (5° S–5° N, 150°–90° W) SST anomaly index ($T_{E}$) and the equatorial Pacific thermocline depth anomalies ($\left[ h \right]$) (Fig. 1). Here the 17 °C isotherm depth is used as a proxy of thermocline depth instead of 20 °C isotherm in order to avoid an outcropping of the 20 °C isotherm to the surface. The phase-space trajectories of ($T_{E}$(t), $\left[ h \right]\left( t \right)$) (see also Fig. 2 of Timmermann et al.^5^) revealed the apparent amplitude- and phase-asymmetries between El Niño and La Niña, respectively. The probability density distribution is not symmetric but skewed toward to positive $T_{E}$ and negative $\left[ h \right]$. Similarly, the trajectories of ($T_{E}$(t), $\left[ h \right]\left( t \right)$) tend to exhibit a far-reaching shape toward positive $T_{E}$ and negative $\left[ h \right]$, indicating a stronger warm phase and a greater oceanic heat content discharge (i.e., shallow thermocline depth) compared to its counterpart^10^. This asymmetric feature provides an easier transition from warm to cold events, compared to the opposite case^27^, as observed. This is because the larger heat discharge associated with the large positive $T_{E}$ produces a strong negative SST tendency through vertical thermal advection; thus, this initiates a cold event more effectively^4^. This occurrence is therefore also directly related to the transition asymmetry^28,29^. Furthermore, the length between two adjacent dots during the warm phase ($T_{E}$ > 0), that indicates the rate of change ($T_{E}$, $\left[ h \right]$), was overall longer than that during the cold phase ($T_{E}$ < 0), which represents the duration asymmetry^12^.

It has been proposed that the positively skewed $T_{E}$ is caused by a combination of oceanic nonlinear thermal advection due to NDH (i.e., oceanic eddy thermal flux)^15,30,31^, an asymmetric atmospheric response to the ENSO-induced SSTA^25,28,32,33^, and a state-dependent noise-induced instability^19,20,24^. The asymmetry in the duration/transition of El Niño and La Niña has been linked to the asymmetric impact of the Indian Ocean heat capacitor effect, where warming of the Indian Ocean accompanied by El Niño reinforces a quick termination (although this rarely occurs during a La Niña event)^12,34,35^. Other hypotheses put forward to explain the duration-asymmetry include (i) nonlinear air-sea coupling^32^, (ii) asymmetry in the delayed negative feedback with respect to the reflective oceanic Kelvin wave^29,36^, or (iii) a strong meridional seasonal marching of the western Pacific surface winds (which is a quick terminator of El Niño but not of La Niña)^37^.

### Nonlinear recharge oscillator model

Following previous studies on the role of NDH^15,30,31^ in setting amplitude asymmetry^15^, we implemented NDH in the ROM, especially the dominant zonal and vertical NDHs^15,31^. Using the formulation of Jin et al.^6^, the zonal and vertical components of NDH can be represented by combinations of $T_{E}$ and $\left[ h \right]$, as follows:

2 $$\left\langle {NDH} \right\rangle_{E} \approx \beta_{1} T_{E} T_{E} + \beta_{2} T_{E} \left[ h \right].$$

Here, $\beta_{1} = \frac{{2\delta \mu_{a} \beta_{us} }}{L} + \frac{1}{{H_{s} }}\beta_{w} \mu_{a} \left\{ {1 - \frac{1}{2}\alpha_{h} \mu_{a} \beta_{h} } \right\}$ and $\beta_{2} = \frac{{2\delta \beta_{uh} }}{L} - \frac{1}{{H_{s} }}\alpha_{h} \beta_{w} \mu_{a}$, where $\mu_{a}$ is a sensitivity factor of the zonal surface wind stress against SSTA; $\beta_{us}$, $\beta_{uh}$,$\beta_{w}$, and $\beta_{h}$ are the sensitivity factors of zonal current against zonal wind stress and thermocline depth, anomalous upwelling against zonal wind stress, and the equatorial zonal slope of thermocline depth anomalies against zonal wind stress anomaly, respectively; $\alpha_{h}$ is the sensitivity factor of the anomalous subsurface temperature anomaly against thermocline depth change; $L$ and $H_{s}$ are the ocean basin length and ocean surface layer depth, respectively.; and $\delta$ is a scaling factor for the zonal gradient of mean SST within the eastern box. Derivations of Eq. (2) are presented in the “Methods” section and also found in Kim et al.^38^. State-dependent (multiplicative) noise can serve as a stochastic nonlinearity, as can be easily demonstrated by introducing logarithmic variables. Here we implement multiplicative noise, in which the stochastic noise amplitude is enhanced only when $T_{E} > 0$, which is qualitatively consistent with observations^20^. Finally, the nonlinear ROM can be represented as

3a $${\raise0.7ex\hbox{${d T_{E} }$} \!\mathord{\left/ {\vphantom {{d T_{E} } {dt}}}\right.\kern-\nulldelimiterspace} \!\lower0.7ex\hbox{${dt}$}} = I_{BJ} T_{E} + F\left[ h \right] + \beta_{1} T_{E}^{2} + \beta_{2} T_{E} \left[ h \right] + \sigma_{1} \left( {1 + BH\left( {T_{E} } \right)T_{E} } \right)\xi_{1} \left( t \right),$$

3b $${\raise0.7ex\hbox{${d\left[ h \right]}$} \!\mathord{\left/ {\vphantom {{d\left[ h \right]} {dt}}}\right.\kern-\nulldelimiterspace} \!\lower0.7ex\hbox{${dt}$}} = - \varepsilon \left[ h \right] - \alpha T_{E} + \sigma_{2} \xi_{2} \left( t \right),$$

where $$\xi_{1}$$, and $$\xi_{2}$$ are independent Gaussian white noise with zero mean and unit variance, and $$\sigma_{1}$$ and $$\sigma_{2}$$ are the amplitudes of noise. The noise has a random part and a state-dependent part, and $$H\left( x \right)$$ is a Heaviside step function, which has a value of one if the argument positive and zero otherwise. From the observed time series of $$T_{E}$$ and $$\left[ h \right]$$, we estimated ROM coefficients ($$I_{BJ}$$, $$F$$, $$\varepsilon$$, $$\alpha , \beta_{1} , \beta_{2} , B$$) via a least-square fitting of $$T_{E}$$ and $$\left[ h \right]$$ tendencies against $$T_{E}$$ and $$\left[ h \right]$$^39^. Although NDH provides a physical meaning on the quadrature terms in (3a), we do not advocate it as the only nonlinear process in ENSO system. Moreover, as ROM coefficients were statistically computed from the observations, other deterministic nonlinear processes were empirically fitted on quadrature terms of either $$\beta_{1}$$ or $$\beta_{2}$$. The amplitudes of noise ($$\sigma_{1} ,$$$$\sigma_{2}$$) and $$B$$ were estimated as the standard deviation of the residual from ROM coefficient fitting. Thus, all coefficients (see “Methods” for their values) were empirically computed and were not retrieved from a separated physical quantity.

### Probabilistic nonlinear model (PROM)

All possible solutions of ROM (Eq. 3) can be expressed as probability density functions (PDFs). To describe the evolution of the probability distribution the 2-dimensional Langevin equation can be translated into a Fokker–Planck recharge oscillator model (PROM). Unlike the ensemble method in previous studies^40,41^, the PROM directly solves for the PDF of the stochastic dynamical system, and it has thus been used to explore noise-perturbed ROM system in recent years^19,21,23,25^ (see “Methods” for the details). First, the two-dimensional Langevin equation in $\left( {T,h} \right)$ space was derived from the nonlinear ROM in Eq. (3) as follows,

4a $$\dot{T} = f_{1} \left( {T,h} \right) + D_{1} G\left( T \right)\xi_{1} \left( t \right),$$

4b $$\dot{h} = f_{2} \left( {T,h} \right) + D_{2} \xi_{2} \left( t \right),$$

where $f_{1} \left( {T,h} \right) = a_{11} T + a_{12} h + \beta_{1} T^{2} + \beta_{2} Th$, $f_{2} \left( {T,h} \right) = a_{21} T + a_{22} h$, $D_{1} = \sigma_{1}$, $D_{2} = \sigma_{2}$ and $G\left( T \right) = \left[ {1 + BH\left( T \right)T} \right]$. $f_{i} \left( {T,h} \right) \left( { i = 1,2} \right)$ is a drift term and the noise amplitude $D_{i} \left( { i = 1,2} \right)$ takes on the form of a diffusion term in the probability. To simplify the notation we replaced parameters used in Eq. (3), ($I_{BJ}$, $F$, − $\varepsilon$, − $\alpha$) by ($a_{11}$, $a_{12}$, $a_{21}$, $a_{22}$) and $T_{E}$ and $\left[ h \right]$ by $T$ and $h$, respectively. We adopt the Stratonovich formulation for the stochastic system. Assuming that stochastic processes can be represented by Gaussian white noise, the FPE is a considerably effective tool to describe the temporal evolution of PDF of the original Langevin system. Therefore, Eq. (4) is converted to a 2-dimensional FPE with state-dependent diffusion (i.e., PROM) for the probability density $P$, as follows,

5 $$\begin{aligned} \frac{\partial P}{{\partial t}} & = - \frac{\partial }{\partial T}\left[ {\left( {f_{1} + \frac{1}{2}D_{1}^{2} G\frac{\partial G}{{\partial T}}} \right)P} \right] - \frac{\partial }{\partial h}\left[ {f_{2} P} \right] + \frac{1}{2}D_{1}^{2} \frac{{\partial^{2} }}{{\partial T^{2} }}\left[ {G^{2} P} \right] + \frac{1}{2}D_{2}^{2} \frac{{\partial^{2} P}}{{\partial h^{2} }} \\ & = - \left( {a_{11} + a_{22} + g_{DN} + g_{SN} } \right)P - \left( {a_{11} T + a_{12} h + f_{DN} + f_{SN} } \right)\frac{\partial P}{{\partial T}} \\ & \quad - \left( {a_{21} T + a_{22} h} \right)\frac{\partial P}{{\partial h}} + \frac{1}{2}\sigma_{1}^{2} \left( {1 + k_{SN} } \right)^{2} \frac{{\partial^{2} P}}{{\partial T^{2} }} + \frac{1}{2}\sigma_{2}^{2} \frac{{\partial^{2} P}}{{\partial h^{2} }}, \\ \end{aligned}$$

where $g_{DN} = 2\beta_{1} T + \beta_{2} h$; $f_{DN} = \beta_{1} T^{2} + \beta_{2} Th$; $g_{SN} = - \frac{1}{2}\sigma_{1}^{2} \left[ {B^{2} H\left( T \right)^{2} + B^{2} \delta \left( T \right)^{2} T^{2} + 3B^{2} \delta \left( T \right)H\left( T \right)T + B\delta \left( T \right)} \right]$; $f_{SN} = - \frac{3}{2}\sigma_{1}^{2} B\left[ {1 + BH\left( T \right)T} \right]\left[ {\delta \left( T \right)T + H\left( T \right)} \right]$; and $k_{SN} = BH\left( T \right)T$. The subscript $DN$ and $SN$ denote the deterministic and stochastic nonlinearity (multiplicative noise contributions), respectively, and $\delta \left( T \right)$ is the Dirac delta function. In the second line of Eq. (5), the first term describes the damping (growth) rate of P, and the second and third terms represent the advection toward the $T$ and $h$ directions with velocities (or nonlinear force) $f_{1} \left( {T,h} \right)$ and $f_{2} \left( {T,h} \right)$, respectively. The fourth and fifth terms capture the diffusion of the PDF induced by noise. As seen in Eq. (5), the damping rate and advection velocity become asymmetric in $\left( {T,h} \right)$ space due to the nonlinear parameters, $\beta_{1}$ and $\beta_{2}$, and state-dependent noise B.

### Time-dependent solutions of PROM

The time evolutions of PROM for the linear version [i.e., $\left( {\beta_{1} , \beta_{2} ,B} \right) = \left( {0,0,0} \right)$ of Eq. (5)] and the nonlinear version were computed using two different initial conditions: an El Niño onset case $\left( {T = 0K,h = 10m} \right)$ and a La Niña onset case $\left( {T = 0K,h = - 10m} \right)$. The corresponding initial PDFs were set as a 2-dimensional Dirac delta function centered on the El Niño and La Niña onset cases, respectively. The time integration of Eq. (5) was then performed (see “Methods” for integration method).

**Figure 2:**
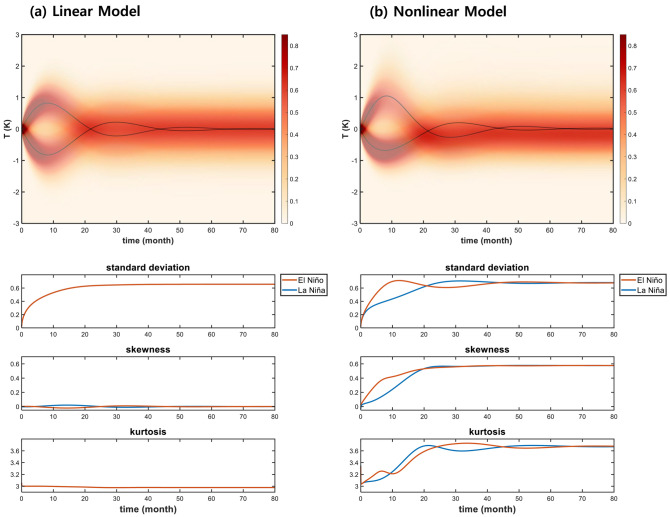
Time evolution of probability density functions. (**a**) Time evolution of PDF with its mean, standard deviation, skewness, and kurtosis obtained from the FPE of linear ROM. Black line and shading of $T$ indicate mean (unit: K) and probability density function (unit: 1), respectively. Red and Blue lines indicate results from warm and cold initial conditions, respectively. (**b**) As in (**a**), except for nonlinear ROM. Units for standard deviation, skewness, and kurtosis are normalized non-dimensional units.

As shown in Fig. 2a, the solution of the Fokker–Planck model of the linear ROM produces a damped oscillatory feature initially, and then it converges to a stationary Gaussian PDF, regardless of the initial conditions. The oscillatory features perturbed by the El Niño and La Niña onset conditions are symmetric. Therefore, the time evolution of the standard deviation and the kurtosis for the El Niño and La Niña onset cases are also identical. The standard deviation increases in an oscillatory regime and then stabilizes after reaching its maximum in the stationary regime, and kurtosis is strongly damped in the oscillatory regime, but it then retains its value in the stationary regime. No skewness is observed.

The nonlinear PROM produces prominent asymmetric features during the oscillatory phase (Fig. 2b). The time evolutions of the standard deviation, skewness, and kurtosis for El Niño and those for La Niña onset cases are all different, although they merge in the stationary regime without difference. Interestingly, the standard deviation in the stationary regime obtained from the linear PROM and those from the nonlinear PROM are almost identical, while skewness and kurtosis are different.

A time integration of PROM showed already some basic features. However, the solutions depended on the initial conditions^21^. Therefore, a more general framework is required to better understand the fundamental features of linear and nonlinear PROMs.

### Eigensolutions of PROM

To obtain a more generalized solution, we apply a variable separation method, where the $P\left( {T,h,t} \right)$ was written as a product of a time dependent function ($e^{{\lambda_{n} t}}$), and a phase-space-dependent function ($\psi_{n} \left( {T,h} \right)$), as follows:

6 $$P\left( {T,h,t} \right) = \mathop \sum \limits_{n = 0}^{\infty } \psi_{n} \left( {T,h} \right)e^{{\lambda_{n} t}} .$$

It should be noted that $\psi$ shall not be directly considered as a probability density, but more as a structure function in phase-space that captures major characteristics of the ENSO model.

On substituting Eq. (6) into Eq. (5), we obtain the eigenvalue problem:

7 $$L_{FP} \psi_{n} \left( {T,h} \right) = \lambda_{n} \psi_{n} \left( {T,h} \right),$$

where $L_{FP} = - \left( {a_{11} + a_{22} + g_{DN} + g_{SN} } \right) - \left( {a_{11} T + a_{12} h + f_{DN} + f_{SN} } \right)\frac{\partial }{\partial T} - \left( {a_{21} T + a_{22} h} \right)\frac{\partial }{\partial h} + \frac{1}{2}\left[ {\sigma_{1}^{2} \left( {1 + k_{SN} } \right)^{2} \frac{{\partial^{2} }}{{\partial T^{2} }} + \sigma_{2}^{2} \frac{{\partial^{2} }}{{\partial h^{2} }}} \right]$. Therefore, the solutions of PROM can be represented by the eigenfunction $\psi_{n} \left( {T,h} \right)$ and the eigenvalue $\lambda_{n}$ as a discrete eigenvalue spectrum^42^. It should be noted that $L_{FP}$ is a non-Hermitian matrix, and thus the eigenfunctions are not orthogonal. If a drift potential, ${\varvec{U}}$, exists such that $f_{i} = - \frac{{\partial {\varvec{U}}}}{{\partial x_{i} }}$, then $L_{FP}$ can be transformed as a Hermitian (self-adjoint system). In this case the FPE becomes a Schrödinger-type equation^21^. However, our 2-dimensional nonlinear system has no explicit drift potential; therefore, the nonlinear PROM cannot be transformed into a self-adjoint system.

**Figure 3:**
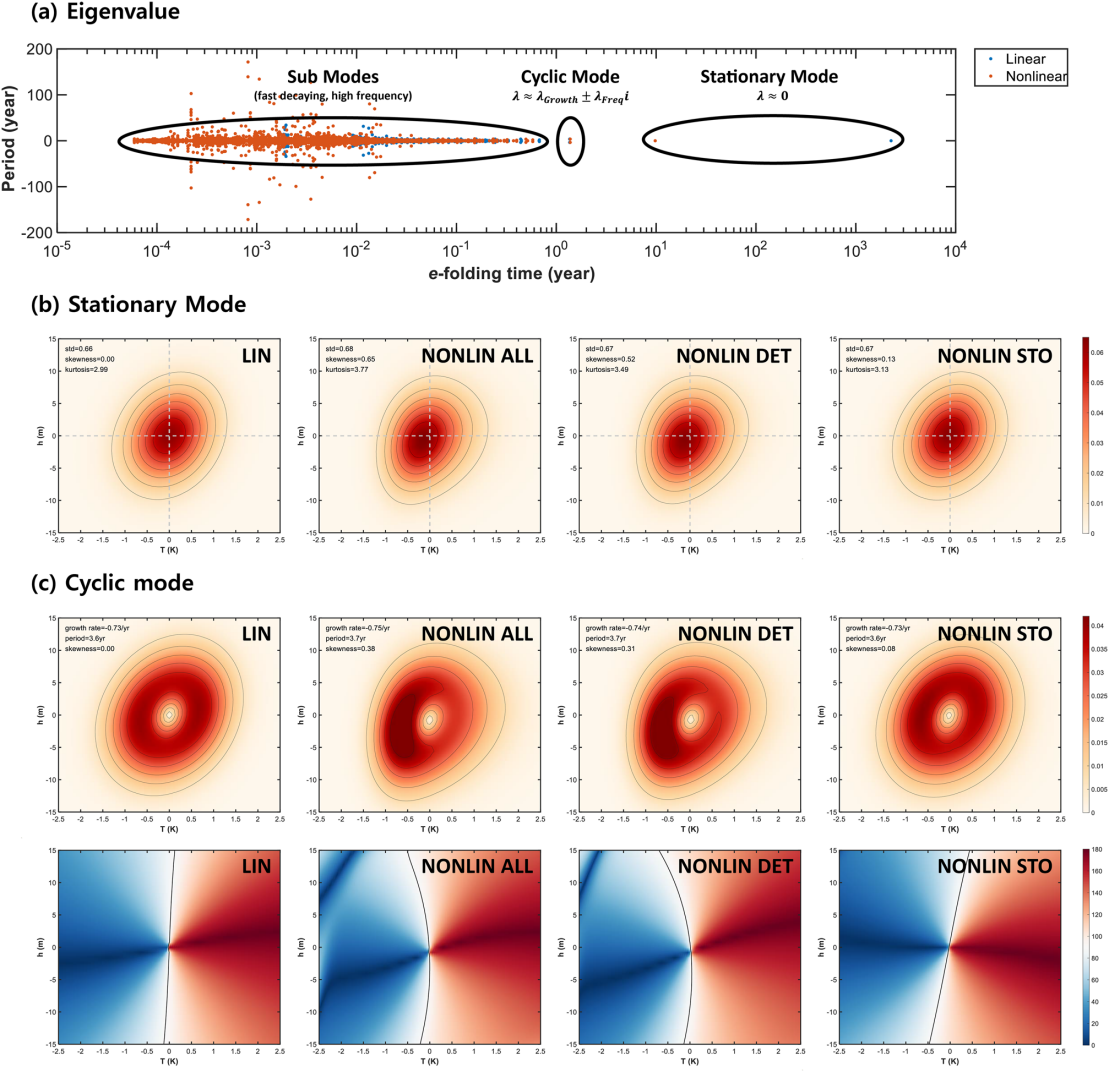
Eigenmode solution for linear and nonlinear PROM. (**a**) Eigenvalues distribution with respect to period (year) and e-folding (year) obtained from FPE of linear ROM (blue dots) and nonlinear ROM (red dots). (**b**) Probability density amplitude ($\sqrt {\psi_{r} \left( {T,h} \right)^{2} + \psi_{i} \left( {T,h} \right)^{2} }$) of stationary mode for linear and nonlinear ROM. Here LIN, NONLIN ALL, NONLIN DET and NONLIN STO denote linear, nonlinear, deterministic nonlinear and stochastic nonlinear (multiplicative noise) model, respectively (see Supplementary Information for equations of each model). The std (standard deviation) and skewness are obtained from $T$. (**c**) Same as (**b**), but for cyclic mode (upper panel) and its phase distribution ($\phi \left( {T,h} \right))$ (lower panel), which characterizes the temporal evolution in phase-space. Units for probability density are %/100. Thick black line in phase distribution indicates 90° phase.

The eigenmode solution was obtained by discretizing the Fokker–Planck operator ($L_{FP}$) into the matrix form (see “Methods” for the detail). The solution of $\lambda_{n}$ had two cases: only real numbers or real and imaginary numbers; here the real and imaginary numbers indicate the growth rate and frequency, respectively. The growth rate and frequency are converted to an e-folding time, $1/Re\left( {\lambda_{n} } \right)$ and period, $2\pi /Im\left( {\lambda_{n} } \right)$, respectively and plotted in Fig. 3a.

The growth rates obtained from both the linear and nonlinear PROMs were all negative (see Fig. 3a), with the exception of one obtained from the nonlinear PROM, which showed a positive growth rate. This positive growth mode is physically unrealistic as the corresponding fixed point $\left( {T = - 3.56,{ }h = 127.76} \right)$ was beyond a realistic range. Although the nonlinear PROM may have a specific domain that shows realistic oscillatory behavior analogous to the maximum potential intensity of ENSO^17^, the positive growth rate mode was beyond of this domain.

Based on their physical characteristics, the eigensolutions were separated in three modes: “stationary”, “cyclic”, and strongly damped “sub” modes (see Fig. 3a). Among these, the stationary mode was the least damped mode and the eigenvalue had only real numbers ($\lambda = - 4.43 \times 10^{ - 4}$ for the linear PROM and $\lambda = - 3.36 \times 10^{ - 2}$ for the nonlinear PROM in units of year^−1^), which indicates an absence of oscillatory features. For example, the stationary mode corresponds to the empirical probability distribution obtained from the histogram of ENSO index. The corresponding probability density distribution (PDD) from the linear PROM (Fig. 3b) shows a symmetric Gaussian distribution, of which the maximum is located at the zero point ($T = 0, h = 0$) (first panel of Fig. 3b), where the zero point corresponds to the mean state of this system or a fixed point $\left( {\frac{\partial P}{{\partial t}} = 0} \right)$, and the PDD resembles a stable focus in dynamical systems’ theory. However, the PDD from the nonlinear PROM shows an asymmetric feature resembling the observed PDD, where $T$ is positively skewed and $h$ is negatively skewed (second panel of Fig. 3b). As the highest density is found at the zeros in the linear PROM and at near zeros in the nonlinear PROM, and the PDD diffuses outward, we could argue that the damping and diffusion terms of Eq. (5) play an important role in inducing this stationary mode. The standard deviation of $T$ in this mode was 0.66 for linear PROM and 0.68 for nonlinear PROM (observed standard deviation $\approx$ 0.81), indicating that the variance of SSTA is mainly determined by the linear stability. Similarly, standard deviations from the deterministic nonlinearity only PROM (third panel of Fig. 2b) and the stochastic nonlinearity only PROM (fourth panel of Fig. 2b) were almost identical (0.67). The small difference between the standard deviations of the linear and nonlinear PROMs could be attributed to the difference in $a_{11}$ and the noise amplitudes, $D_{1}$, and $D_{2}$. The difference between the modeled (Fig. 3) and the observed standard deviations is likely due to the lack of an annual cycle and/or the rather simplified formulation. Meanwhile, the skewness of $T$ (i.e., the normalized third order moment) was 0 for the linear PROM and 0.65 for the nonlinear PROM, and 0.84 in the observations presented in Fig. 1. The skewness from the stochastic nonlinearity only PROM is 0.13. However, for a fair comparison, we actually re-estimated the parameters for each PROM version independently; and with these independently computed parameters, the deterministic nonlinear PROM (0.52) still produced larger skewness than the stochastic nonlinear PROM (0.34). Thus, the positively skewed SSTA can be attributed to the nonlinear process, especially by the deterministic nonlinearity.

The second least damped modes of both the linear and nonlinear PROMs are a “cyclic mode”, of which the eigenvalue is a pair of complex numbers: ($\lambda = \lambda_{r} \pm i\lambda_{i} = - 0.734 \pm 1.726 i$ for linear PROM and $\lambda = - 0.751 \pm 1.720i$ for the nonlinear PROM; units are year^−1^), corresponding to an oscillatory mode. Also, the eigenfunction has a conjugate pair, $\psi = \psi_{r} \pm i\psi_{i}$, and these conjugate solution pairs can be combined as follows:

8 $$\psi_{osc} \left( {T,h,t} \right) = 2e^{{\lambda_{r} t}} \sqrt {\psi_{r} \left( {T,h} \right)^{2} + \psi_{i} \left( {T,h} \right)^{2} } cos\left[ {\lambda_{i} t + \phi \left( {T,h} \right)} \right],$$

where the phase is $\phi \left( {T,h} \right) = arccos\left( {\frac{{\psi_{r} }}{{\sqrt {\psi_{r}^{2} + \psi_{i}^{2} } }}} \right)$. The combined function exhibits oscillatory behavior with period = $\frac{2\pi }{{\lambda_{i} }}$ and growth rate = $\lambda_{r}$. This mode could be regarded as a stochastically forced recharge oscillator mode, with a 3.6-year period and − 0.73 (− 0.75) year^−1^ e-folding damping for both the linear and nonlinear PROMs. It should be noted that the stationary modes are corresponding to the equilibrium state of time-dependent solutions of PROM, while the oscillatory features in the early period of time-dependent solutions are representing the cyclic mode (see Fig. 2).

The cyclic mode is obviously related to the FPE advection terms in Eq. (5). The first and second panels of Fig. 3c, referring to the linear and nonlinear PROM, respectively, show the probability density amplitude ($\sqrt {\psi_{r} \left( {T,h} \right)^{2} + \psi_{i} \left( {T,h} \right)^{2} }$). The probability density amplitude of the linear PROM is uniformly distributed along an ellipse, which closely resembles a limit cycle. However, the probability density amplitude of nonlinear PROM has an oval shape with a small eccentricity, which represents the non-symmetric oscillation of ENSO^10^.

The phase distribution also supports the difference between the oscillatory features of the linear and nonlinear PROMs. The expected duration of the warm event ($T > 0$) is approximately 6 months shorter than that of the cold event ($T < 0$) in the cyclic mode of nonlinear PROM (Fig. 3c). This difference is also observed in the stationary mode of the nonlinear PROM; however, it is relatively shorter (~ 2 months) than that in the oscillatory mode (not shown). Equation (8) further confirms that the difference in the temporal behavior can be attributed to the difference in phase, as the phase is determined by the eigen function rather than the eigenvalue.

Differences in growth rate and period between the nonlinear deterministic PROM (third panel of Fig. 3c) and the nonlinear stochastic PROM (fourth panel of Fig. 3c) are marginal, but the skewness are quite distinct. The cyclic mode from the nonlinear stochastic PROM is actually similar to that from the linear PROM, while that from the nonlinear deterministic PROM is almost identical to the fully nonlinear PROM (including deterministic nonlinearities and state-dependent noise). It indicates that the asymmetric oscillatory behavior of this system is mainly driven by the deterministic nonlinearity.

### Evolution of statistical moments

**Figure 4:**
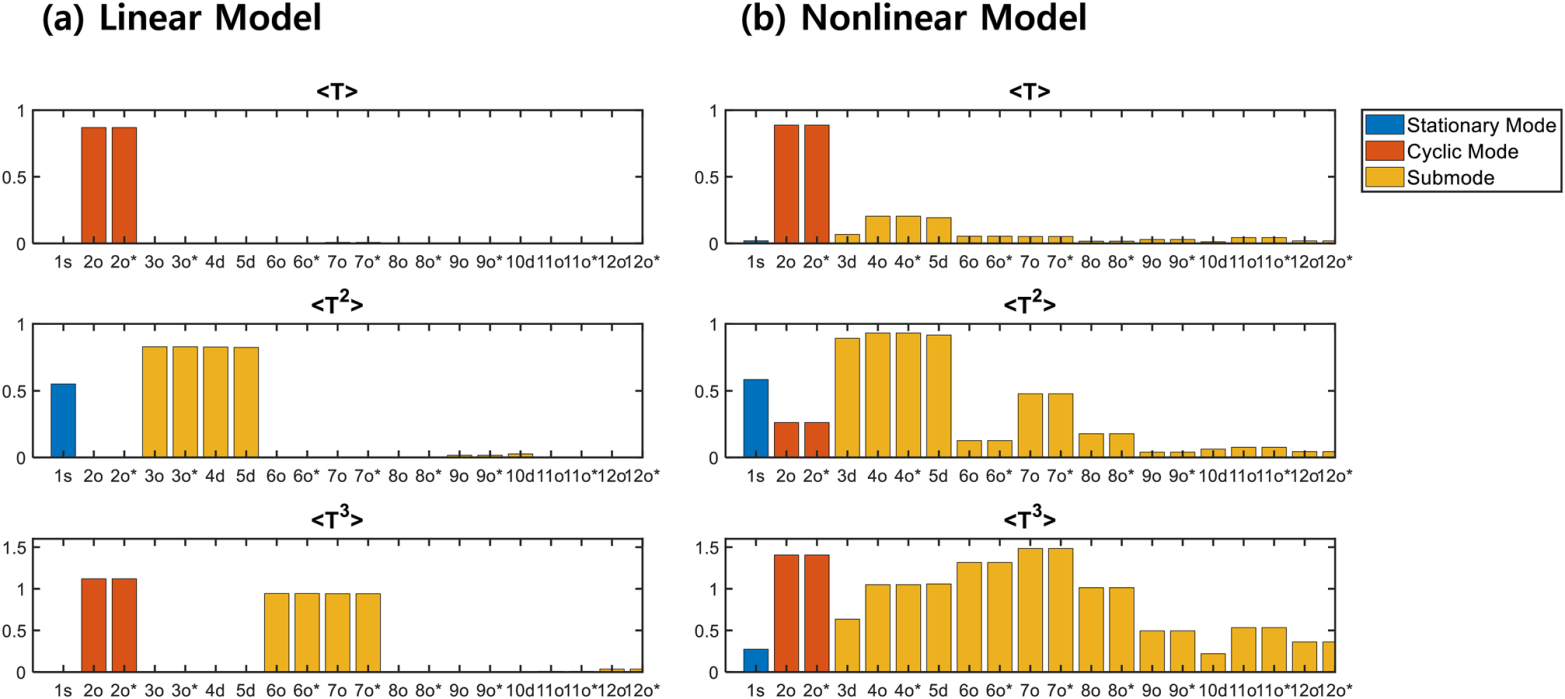
Projection of PROM eigenmodes to statistical moments. (**a**) Projection by each mode on $T, T^{2}$, and $T^{3}$ obtained from FPE of the linear ROM. (**b**) As in (**a**) but for the nonlinear ROM.

To link the time evolution of statistical moment of the PDF and eigenmode, we computed the projection of each eigenmode on each statistical moment. It should be noted that the original statistical moments cannot be fully reconstructed by the eigenmode projection because of the nonorthogonality between eigenmodes. The corresponding mathematical expression is,

9 $$\left\langle {T^{k} } \right\rangle \left( t \right) = \mathop \sum \limits_{n = 1}^{\infty } \sqrt {[\left\langle {T^{k} } \right\rangle_{n}^{real} ]^{2} + [\left\langle {T^{k} } \right\rangle_{n}^{imag} ]^{2} } e^{{\lambda_{n}^{r} t}} cos\left( {\lambda_{n}^{i} t + \phi_{n} } \right),$$

where *n* indicates the mode and $\phi_{n}$ = $tan^{ - 1} \left( {\frac{{\left\langle {T^{k} } \right\rangle_{n}^{imag} }}{{\left\langle {T^{k} } \right\rangle_{n}^{real} }}} \right)$ (see “Methods” for detailed derivations). Figure 4 shows the projection of each mode, i.e., $\sqrt {[\left\langle {T^{k} } \right\rangle_{n}^{real} ]^{2} + [\left\langle {T^{k} } \right\rangle_{n}^{imag} ]^{2} }$. For the linear PROM, all the eigenfunctions is either symmetric or antisymmetric (See Supplementary Fig. 1). Hence, the odd (even) statistical moment can be solely determined by the antisymmetric (symmetric) function ($\left\langle {T^{k} } \right\rangle = \smallint T^{k} \psi \left( T \right)dT$). The amplitude of $\left\langle T \right\rangle$ is solely determined by the cyclic mode (n = 2), and thus the temporal evolution feature of $T$ is determined by the cyclic mode (the fluctuation of the PDF can be seen in Fig. 2a), whereas the amplitude of $\left\langle {T^{2} } \right\rangle$ is mainly determined by the stationary mode (n = 1) and sub-modes (mainly n = 3 ~ 5) and the cyclic mode does not produce it. Both the stationary mode and sub-modes (n = 3 ~ 5) have symmetric eigenfunctions for $T$ (i.e., $\psi \left( T \right) = \smallint \psi \left( {T,h} \right)dh$), whereas the cyclic mode has an antisymmetric eigenfunction (see Supplementary Fig. 1), which does not contribute to the amplitude of $\left\langle {T^{2} } \right\rangle$. In contrast, $\left\langle {T^{3} } \right\rangle$ is determined by antisymmetric eigenfunctions: the cyclic mode (n = 2) and the sub-modes (n = 6, 7).

In the nonlinear PROM, none of the eigenfunctions are simply either symmetrical or asymmetrical function, due to the nonlinear dynamics (see Supplementary Fig. 1) which leads to the interaction of statistical moments. The broken symmetry causes entrainment of the extra eigenmodes, which is not the case in the linear PROM. Thus, each moment is determined by a complex combination of eigenfunctions. Therefore, not only the cyclic mode (n = 2), but also several sub-modes contribute to the evolutionary feature of $T$: the cyclic mode also contributes $\left\langle {T^{2} } \right\rangle$; and $\left\langle {T^{3} } \right\rangle$ is determined by almost all modes, even by the stationary mode.

The power spectrum of each statistical moment further demonstrates the entrainment effect of the sub-modes through nonlinear dynamics (Supplementary Fig. 2). The spectrum of $\left\langle T \right\rangle$ exhibits a spectral peak that is pronounced in the cyclic mode (n = 2) and smoothly decays toward a higher frequency in both linear and nonlinear models. However, there are some weak anomalies in the higher frequency band in the nonlinear model, that are related to sub-modes and higher order nonlinear resonances. These anomalies that feature in the nonlinear model also appear in the standard deviation and skewness spectra, particularly in the lower frequency band, mainly due to the cyclic mode (n = 2) and the sub-modes (n = 3 ~ 4). The spectral density of skewness in the nonlinear model is significantly larger than that in the linear model.

## Discussion

In this study, the original linear ROM was expanded to a nonlinear PROM by including deterministic and stochastic nonlinearities. This nonlinear PROM is different from other previously modified ROMs or delayed oscillator models presented in previous studies^19,25,29,32,43^, where empirical parameterizations rather than dynamical parameterizations have been adopted. Furthermore, to provide a more complete phase-space perspective, rather than a local or initial-condition dependent picture, the probability density was directly computed using an eigen analysis; thus it was possible to compare all possible probability distributions of the ENSO system derived from the nonlinear ROM to the observed probability distribution. The nonlinear PROM well mimics the observed nonlinear features of ENSO and shows asymmetries in amplitude, duration, and oscillatory behavior. Moreover, the eigenanalysis of the nonlinear PROM elegantly reveals features about the evolution of the probability distribution and its moments that are not as evident by just analyzing the Langevin equation of the nonlinear stochastic ROM. With the PROM, the relative role of deterministic and stochastic nonlinearities in driving asymmetric features of ENSO can be assessed both qualitatively and quantitatively, in terms of not only basic statistics (variance, skewness) but also temporal behaviors in a phase space. Our results indicate that the deterministic nonlinearity is more important to induce the observed ENSO asymmetry than the stochastic nonlinearity related to the state-dependent noise. In summary, the decomposition of intrinsic probability density distribution features of ENSO physics, including stationary, cyclic, and other sub-modes, provide elegant and new insights into the nature of ENSO’s temporal complexity, and the method used in this study could thus become a useful diagnostic tool to further study the complexity of ENSO in complex climate model simulations.

Because of two spatial degrees of freedom, PROM could not address the spatial complexity of ENSO such as the spatial difference in the maximum loading of SST anomaly between El Niño and La Niña^44^, and two types of El Niño (i.e., Central and Eastern Pacific types^45,46^). Some of the other processes driving the temporal ENSO complexity are also missing, such as the annual cycle-ENSO interaction^5^.

## Methods

### Datasets

$T_{E}$ was obtained from the National Oceanic and Atmospheric Administration’s (NOAA) Extended Reconstructed Sea Surface Temperature version 5 (ERSSTv5) dataset^47^ and $\left[ h \right]$ is a merged product of the Simple Ocean Data Assimilation version 2.2.4 (SODAv2.2.4) for 1958–2010^48^ and NCEP Global Ocean Data Assimilation System (GODAS) reanalysis dataset for 2011–2016^49^. Prior to computing the parameters, the climatological annual cycle and linear trends were removed.

### Approximations

The approximations used in the linear ROM^4,6^ were applied to NDH. The zonal component of NDH $$\left( { - u\frac{dT}{{dx}}} \right)$$ is dominant over the far eastern equatorial Pacific^31^. Therefore, as a 2-box approximation, $$\frac{dT}{{dx}}$$ can be represented as $$- \frac{{\delta <T>_{E} }}{L/2}$$, which represents the zonal SST gradient between the center of eastern Pacific and the eastern boundary, where $$\delta$$ indicates the SST decreasing rate between two locations (~ 0.3); $$L$$ is the Pacific basin length; and the subscript *E* represents the equatorial eastern Pacific. The zonal current over the eastern Pacific can be represented as $$\left\langle u \right\rangle_{c} = \beta_{us} \left\langle {\tau_{x} } \right\rangle + \beta_{uh} \left[ h \right]$$ under the 2-box approximation^6^, where the first and second terms represent the wind-driven current and the geostrophic current, respectively, and $$\beta_{us}$$ and $$\beta_{uh}$$ are the corresponding sensitivity parameters. As $$\left\langle {\tau_{x} } \right\rangle$$ = $$\mu_{a} \left\langle T \right\rangle_{E}$$, which indicates the wind stress response against SSTA, and $$\mu_{a}$$ is a sensitivity parameter, the zonal component of NDH becomes $$- u\frac{\partial T}{{\partial x}} \to \frac{{\mu_{a} \beta_{us} }}{L/2}\delta \left\langle T \right\rangle_{E}^{2} + \frac{{\beta_{uh} }}{L/2}\delta \left[ h \right]\left\langle T \right\rangle_{E}$$. The vertical component of NDH $$\left( { - w\frac{dT}{{dz}}} \right)$$ over the equatorial eastern Pacific can be written as $$- \left\langle w \right\rangle_{E} \frac{{<T>_{E} - \left\langle {T_{sub} } \right\rangle_{E} }}{\Delta z}$$, where the subscript *sub* indicates the oceanic subsurface, and $$\Delta z$$ (= $$H_{s}$$) is the ocean surface layer depth. As upwelling velocity, $$\left\langle w \right\rangle_{E} = - \beta_{w} \left\langle {\tau_{x} } \right\rangle$$ = $$- \beta_{w} \mu_{a} \left\langle T \right\rangle_{E}$$ and subsurface temperature $$\left\langle {T_{sub} } \right\rangle_{E} = \alpha_{h} \left\langle h \right\rangle_{E} = \alpha_{h} \left( {\left[ h \right] + \frac{1}{2}\beta_{h} \mu_{a} \left\langle T \right\rangle_{E} } \right)$$, the vertical component of NDH becomes $$- w\frac{dT}{{dz}} \to$$$$\frac{1}{\Delta z}\beta_{w} \mu_{a} \left\{ {1 - \frac{1}{2}\alpha_{h} \mu_{a}^{*} \beta_{h} } \right\}\left\langle T \right\rangle_{E}^{2} - \frac{1}{\Delta z}\alpha_{h} \beta_{w} \mu_{a} \left[ h \right]\left\langle T \right\rangle_{E}$$.

### Coefficient values for ROM

The coefficients used in Eq. (5) are listed in Table 1 where values in (*) indicate coefficients used in the linear ROM, otherwise in the nonlinear ROM.

**Table 1:** Coefficient for linear and nonlinear PROM.

Parameter	Linear component				Nonlinear deterministic		Stochastic forcing		
	$$a_{11}$$$$\left( {{\text{K}}\,{\text{month}}^{ - 1} } \right)$$	$$a_{12}$$ ($${\text{K}}\,{\text{m}}^{ - 1 } \,{\text{month}}^{ - 1}$$)	$$a_{21}$$$$\left( {{\text{m}}\,{\text{K}}^{ - 1 } \,{\text{month}}^{ - 1} } \right)$$	$$a_{22}$$$$\left( {{\text{m}}\,{\text{month}}^{ - 1} } \right)$$	$$\beta_{1}$$$$\left( {{\text{K}}^{ - 1} \,{\text{month}}^{ - 1} } \right)$$	$$\beta_{2}$$$$\left( {{\text{m}}^{ - 1} \,{\text{month}}^{ - 1} } \right)$$	$$\sigma_{1}$$$$\left( {{\text{K}}\,{\text{month}}^{ - 1} } \right)$$	$$B$$$$\left( {{\text{K}}^{ - 1} } \right)$$	$$\sigma_{2}$$$$\left( {{\text{m}}^{ - 1} \,{\text{month}}^{ - 1} } \right)$$
Value	− 0.093	0.021	− 1.02	− 0.028	0.012	0.007	0.218	0.193	1.68
	(− 0.093)	(0.021)	(− 1.02)	(− 0.028)	(0.00)	(0.00)	(0.218)	(0.00)	(1.68)

### Langevin equation and FPE

A Langevin equation is a stochastic differential equation that describes dynamics of a system under random forcing. Originally, it was developed to describe Brownian motion^50^, the random movement of a particle in a fluid. Generalizing the idea, it has been adopted in climate dynamics field. In this adoption the main climate variable of the Langevin equation corresponds to a slowly changing climate state (e.g., SST variability over tropical Pacific) and noise corresponding to weather, a short time scale process^51^. Another equivalent formulation of the Langevin equation is the FPE, which describes the temporal evolution of the probability distribution of the stochastic system. It is identical to ensemble solution for Langevin equation. The explicit formulation of the dynamics of the PDF through the FPE enables us to explore fundamentals of the stochastic system, rather than tracking individual random trajectories.

### Numerical calculation of FPE

The time-dependent FPE (Eq. 5) is computed using the central difference method in [201 × 51] grids on a rectangular domain of $T = \left[ { - 5, 5} \right]$ and $h = \left[ { - 20, 20} \right]$ with Euler method for $\Delta t = 0.01$ month interval. The boundary conditions are set as $p\left( { - 5, h} \right) = p\left( {5,h} \right) = p\left( {T, - 20} \right) = p\left( {T,20} \right) = 0$, and are referred to as absorbing boundaries that resemble the observed range of $\left( {T,h} \right)$ (e.g., Fig. 1). The Dirac delta function in the equation is approximated as $$\delta \left( x \right) = \left\{ {\begin{array}{*{20}c} {1/\left( {2\Delta x} \right), \quad for - \Delta x < x < \Delta x } \\ {0, \quad for\,elsewhere} \\ \end{array} } \right.$$ where $\Delta x$ is grid size. We confirmed that the total probability, ${\iint }p\left( {T,h} \right)dTdh$ is almost conserved as 1 over time (not shown here), indicating that the numerical method is suitable. The sensitivity test for initial condition was carried out by varying $h$ from default initial condition ($T = 0K,h = \pm 10m$), and the overall features of solution (Fig. 2) are unchanged regardless of choice of initial $h$ (not shown here). Similarly, the eigensolution (Eq. 7) is solved by discretizing Fokker–Planck operator ($L_{FP}$) using the central difference method for the same boundary and numerical conditions as in the time-dependent FPE case.

### Eigenmode formulation of statistical moment

The time evolution of the $k$-th statistical moment ($\left\langle {T^{k} } \right\rangle \left( t \right)$) is formulated as the linear sum of eigenmodes (Eq. 9). The statistical moment of the marginal distribution for $T$ is written as $\left\langle {T^{k} } \right\rangle \left( t \right) = {\iint }T^{k} p\left( {T,h,t} \right) dTdh$. Here, the time evolution of PDF ($p\left( {T,h,t} \right)$) can be expanded as $p\left( {T,h,t} \right) = \sum\nolimits_{n = 1}^{\infty } {\psi_{n} } \left( {T,h} \right)e^{{\lambda_{n} t}} = \sum\nolimits_{n = 1}^{\infty } {e^{{\lambda_{n}^{r} t}} } \left[ {cos\left( {\lambda_{n}^{i} t} \right) + isin\left( {\lambda_{n}^{i} t} \right)} \right]\left[ {\psi_{n}^{r} \left( {T,h} \right) + i \psi_{n}^{i} \left( {T,h} \right)} \right]$. Upon substituting the expanded PDF with the statistical moment with respect to only the real part, we get

$$\left\langle {T^{k} } \right\rangle \left( t \right) = \mathop \sum \limits_{n = 1}^{\infty } e^{{\lambda_{n}^{r} t}} cos\left( {\lambda_{n}^{i} t} \right)\left[ {{\iint }T^{k} \psi_{n}^{r} \left( {T,h} \right)dTdh } \right] - e^{{\lambda_{n}^{r} t}} sin\left( {\lambda_{n}^{i} t} \right)\left[ {{\iint }T^{k} \psi_{n}^{i} \left( {T,h} \right)dTdh } \right].$$

Here, the imaginary part is intentionally neglected, as it would be eliminated by its complex conjugate. Using trigonometric identities, the time evolution of the statistical moment becomes

$$\left\langle {T^{k} } \right\rangle \left( t \right) = \mathop \sum \limits_{n = 1}^{\infty } \sqrt {[\left\langle {T^{k} } \right\rangle_{n}^{real} ]^{2} + [\left\langle {T^{k} } \right\rangle_{n}^{imag} ]^{2} } e^{{\lambda_{n}^{r} t}} cos\left( {\lambda_{n}^{i} t + \phi_{n} } \right),$$

where $$\left\langle {T^{k} } \right\rangle_{n}^{real} = {\iint }T^{k} \psi_{n}^{r} \left( {T,h} \right)dTdh,{ }\left\langle {T^{k} } \right\rangle_{n}^{imag} = {\iint }T^{k} \psi_{n}^{r} \left( {T,h} \right)dTdh,{ }\phi_{n} = \tan^{ - 1} \left( {\frac{{\left\langle {T^{k} } \right\rangle_{n}^{imag} }}{{\left\langle {T^{k} } \right\rangle_{n}^{real} }}} \right)$$.

## Supplementary information

Supplementary Information.

## Data Availability

All datasets used in this research can be accessed via the following website: ERSST; https://www1.ncdc.noaa.gov/pub/data/cmb/ersst/v5/netcdf/, SODA; https://coastwatch.pfeg.noaa.gov/erddap/griddap/hawaii_d90f_20ee_c4cb.html GODAS; https://psl.noaa.gov/data/gridded/data.godas.html. The code is available from the authors upon request.
