# Supplementary material for: Fokker–Planck dynamics of the El Niño-Southern Oscillation
Source: Sci Rep. 2020 Oct 1;10:16282. doi: 10.1038/s41598-020-73449-7 (PMC7529818; doi:10.1038/s41598-020-73449-7)
Supplement: Supplementary file 1 — Supplementary Information. [file 41598_2020_73449_MOESM1_ESM.docx]

Supplementary Information for

**Fokker-Planck Dynamics of the El Niño-Southern Oscillation**

Soon-Il An^1^, Soong-Ki Kim^1^, and Axel Timmermann^2,3^

**1** Yonsei University, 50 Yonsei-ro, Seodaemun-gu, Seoul 03722, South Korea

**2** Center for Climate Physics, Institute for Basic Science, Busandaehak-ro 63beon-gil 2 (Jangjeon-dong), Geumjeong-gu, Busan 46241, South Korea

**3** Pusan National University, 2 Busandaehak-ro 63beon-gil 2 (Jangjeon-dong), Geumjeong-gu, Busan 46241, South Korea

**Corresponding Author**: Soon-Il An, [sian@yonsei.ac.kr](mailto:sian@yonsei.ac.kr)

**Supplementary Table 1 | ROM Model Equations**

| Model | Equation |
| --- | --- |
| Linear Model | $\frac{d T_{E}}{dt}=I_{BJ}T_{E}+F\left[ h \right]+\sigma_{1}\xi_{1}(t)$  $\frac{d\left[ h \right]}{dt}=-\varepsilon\left[ h \right]-\alpha T_{E}+\sigma_{2}\xi_{2}(t)$ |
| Nonlinear Model | $\frac{d T_{E}}{dt}=I_{BJ}T_{E}+F\left[ h \right]+\beta_{1}T_{E}^{2}+\beta_{2}T_{E}\left[ h \right]+\sigma_{1}\left( 1+BH\left( T_{E} \right) \right)\xi_{1}\left( t \right)$  $\frac{d\left[ h \right]}{dt}=-\varepsilon\left[ h \right]-\alpha T_{E}+\sigma_{2}\xi_{2}(t)$ |
| Nonlinear Deterministic | $\frac{d T_{E}}{dt}=I_{BJ}T_{E}+F\left[ h \right]+\beta_{1}T_{E}^{2}+\beta_{2}T_{E}\left[ h \right]+\sigma_{1}\xi_{1}\left( t \right)$  $\frac{d\left[ h \right]}{dt}=-\varepsilon\left[ h \right]-\alpha T_{E}+\sigma_{2}\xi_{2}(t)$ |
| Nonlinear Stochastic | $\frac{d T_{E}}{dt}=I_{BJ}T_{E}+F\left[ h \right]+\sigma_{1}\left( 1+BH\left( T_{E} \right) \right)\xi_{1}\left( t \right)$  $\frac{d\left[ h \right]}{dt}=-\varepsilon\left[ h \right]-\alpha T_{E}+\sigma_{2}\xi_{2}(t)$ |

**Supplementary Table 2 | PROM Model Equations**

| Model | In Equation (5) |
| --- | --- |
| Linear Model | $g_{DN}=0$; $f_{DN}=0$; $g_{SN}=0$; $f_{SN}=0$ and $k_{SN}=0$ |
| Nonlinear Model | $g_{DN}=2\beta_{1}T+\beta_{2}h$; $f_{DN}=\beta_{1}T^{2}+\beta_{2}Th$; $g_{SN}=-\frac{1}{2}{\sigma_{1}}^{2}\left[ B^{2}{H(T)}^{2}+B^{2}{\delta(T)}^{2}T^{2}+3B^{2}\delta(T)H\left( T \right)T+B\delta\left( T \right) \right]$; $f_{SN}=-\frac{3}{2}{\sigma_{1}}^{2}B\left[ 1+BH\left( T \right)T \right]\left[ \delta\left( T \right)T+H(T) \right]$; and $k_{SN}=BH\left( T \right)T$ |
| Nonlinear Deterministic | $g_{DN}=2\beta_{1}T+\beta_{2}h$; $f_{DN}=\beta_{1}T^{2}+\beta_{2}Th$; $g_{SN}=0$; $f_{SN}=0$; and $k_{SN}=0$ |
| Nonlinear Stochastic | $g_{DN}=0$; $f_{DN}=0$; $g_{SN}=-\frac{1}{2}{\sigma_{1}}^{2}\left[ B^{2}{H(T)}^{2}+B^{2}{\delta(T)}^{2}T^{2}+3B^{2}\delta(T)H\left( T \right)T+B\delta\left( T \right) \right]$; $f_{SN}=-\frac{3}{2}{\sigma_{1}}^{2}B\left[ 1+BH\left( T \right)T \right]\left[ \delta\left( T \right)T+H(T) \right]$; and $k_{SN}=BH\left( T \right)T$ |


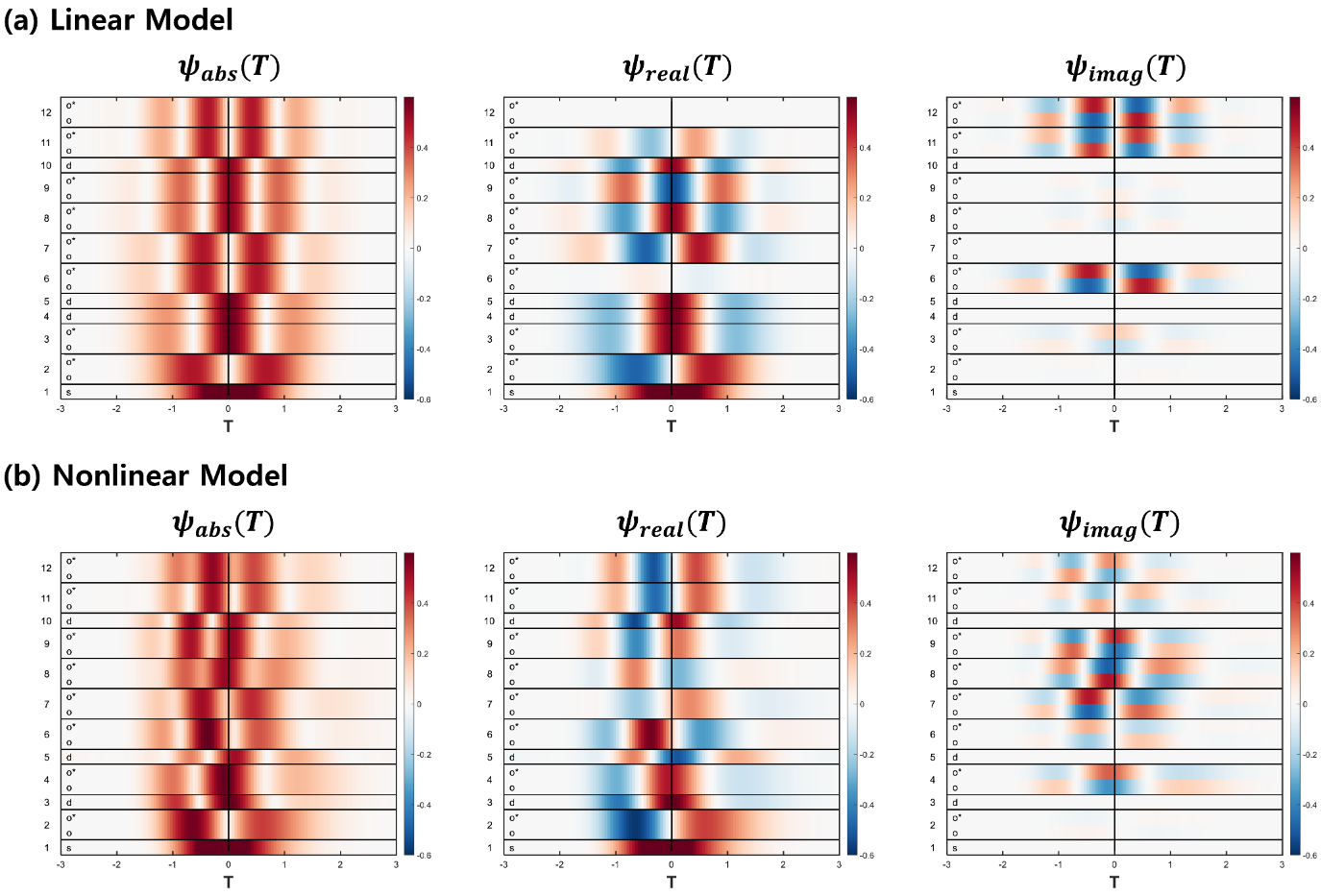


**Supplementary Figure 1 | Eigenfunction solution for linear and nonlinear PROM.** **(a)** The eigenfunction solution for linear ROM’s FPE. The absolute value, real and imaginary part of marginal eigenfunction of $T$ ($\psi(T)=\int\psi(T,h)dh$) is illustrated for 20 number of least damping modes. The *s*, *o* (*o**) and *d* denote stationary, oscillatory and pure decaying mode, respectively. **(b)** As in (a) expect nonlinear ROM’s FPE.


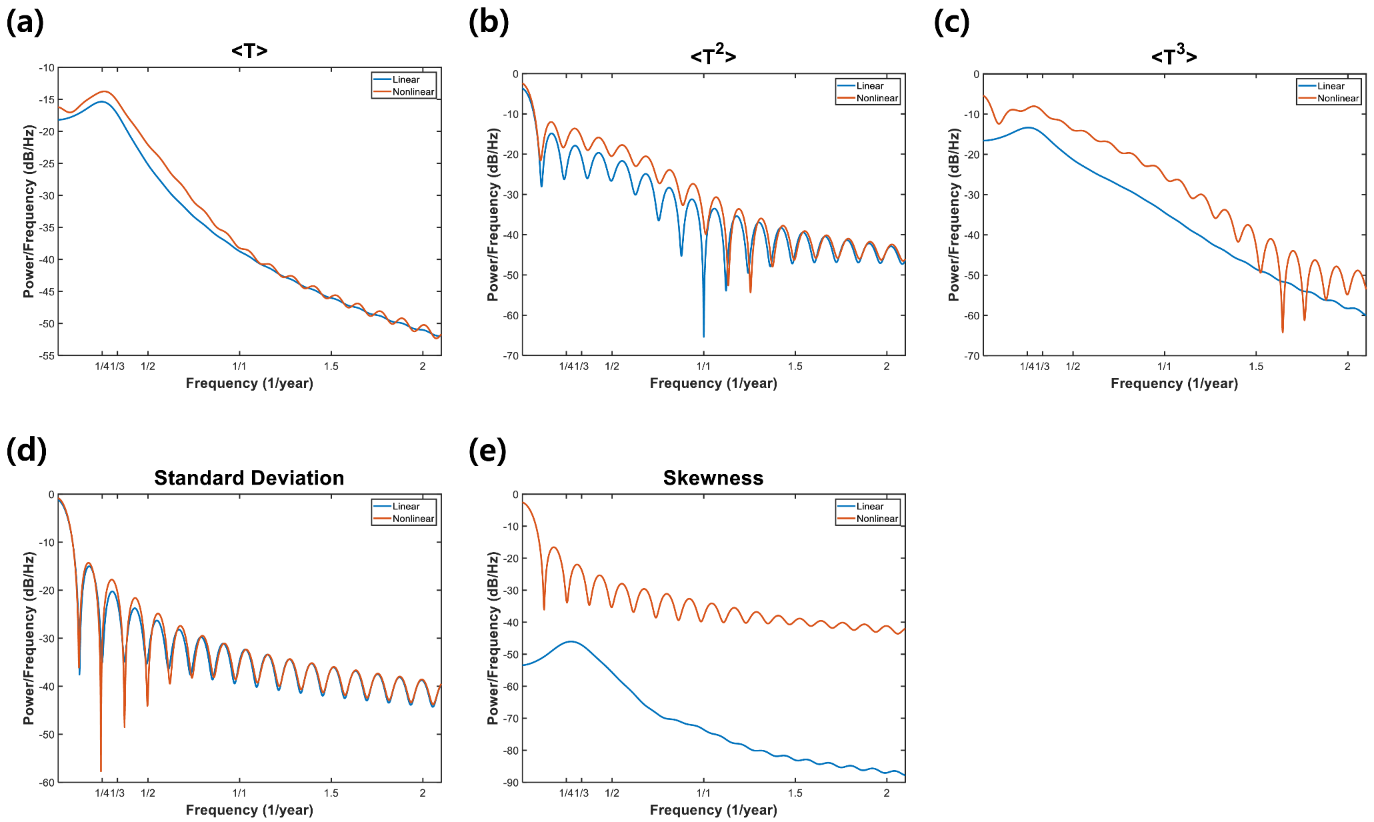


**Supplementary Figure 2 | Power spectrum for statistical moment. (a)** Power spectrum density of time series of $T$ obtained by time integration of FPE. **(b)**, **(c)**, **(d)** and **(e)** As in (a) except $T^{2}$, $T^{3}$, standard deviation and skewness, respectively. Blue and red lines indicate the result from linear and nonlinear ROMs, respectively.
